# Predictors of long-term pain and disability in patients with low back pain investigated by magnetic resonance imaging: A longitudinal study

**DOI:** 10.1186/1471-2474-12-234

**Published:** 2011-10-14

**Authors:** Philip McNee, James Shambrook, E Clare Harris, Miranda Kim, Madeleine Sampson, Keith T Palmer, David Coggon

**Affiliations:** 1 Centre for Defence Imaging and Queen Alexandra Hospital, Portsmouth, UK; 2 Southampton University Hospitals NHS Trust, Southampton, UK; 3 MRC Lifecourse Epidemiology Unit, University of Southampton, Southampton, UK

## Abstract

**Background:**

It is possible that clinical outcome of low back pain (LBP) differs according to the presence or absence of spinal abnormalities on magnetic resonance imaging (MRI), in which case there could be value in using MRI findings to refine case definition of LBP in epidemiological research. We therefore conducted a longitudinal study to explore whether spinal abnormalities on MRI for LBP predict prognosis after 18 months.

**Methods:**

A consecutive series of patients aged 20-64 years, who were investigated by MRI because of mechanical LBP (median duration of current episode 16.2 months), were identified from three radiology departments, and those who agreed completed self-administered questionnaires at baseline and after a mean follow-up period of 18.5 months (a mean of 22.2 months from MRI investigation). MRI scans were assessed blind to other clinical information, according to a standardised protocol. Associations of baseline MRI findings with pain and disability at follow-up, adjusted for treatment and for other potentially confounding variables, were assessed by Poisson regression and summarised by prevalence ratios (PRs) with their 95% confidence intervals (CIs).

**Results:**

Questionnaires were completed by 240 (74%) of the patients who had agreed to be followed up. Among these 111 men and 129 women, 175 (73%) reported LBP in the past four weeks, 89 (37%) frequent LBP, and 72 (30%) disabling LBP. In patients with initial disc degeneration there was an increased risk of frequent (PR 1.3, 95%CI 1.0-1.9) and disabling LBP (PR 1.7, 95%CI 1.1-2.5) at follow-up. No other associations were found between MRI abnormalities and subsequent outcome.

**Conclusions:**

Our findings suggest that the MRI abnormalities examined are not major predictors of outcome in patients with LBP. They give no support to the use of MRI findings as a way of refining case definition for LBP in epidemiological research.

## Background

Low back pain (LBP) has been linked with various abnormalities of the spine detectable on magnetic resonance imaging (MRI), including disc herniation, nerve root impingement, disc degeneration and high intensity zone (HIZ). However, many patients with LBP do not exhibit any of these pathological features, and even when the abnormalities are present, they are not necessarily responsible for the symptom [1]. If risk factors or prognosis for LBP differed importantly according to the presence or absence MRI abnormalities, there could be value in using MRI findings to refine case definitions for the disorder in epidemiological research.

We recently compared clinical presentations and associations with risk factors in patients with LBP who were investigated by MRI, according to whether specified spinal abnormalities were observed. Those with nerve root deviation or compression were more likely to report radiation of their pain to below the knee and associated neurological symptoms in the leg [2]. However, there was no clear difference between cases with and without spinal pathology in associations with either physical or psychological risk factors.

We here report findings when the same LBP patients were followed up at a mean of 22.2 months after their MRI investigation, looking for differences in clinical outcome according to the presence of spinal pathology at baseline. In particular, we focused on the presence of continuing pain and associated disability (including impacts on employment) as outcome measures, and whether, after allowance for treatment, these were predicted by the presence or absence of each of four categories of spinal abnormality.

## Methods

Through the radiology departments at Southampton General Hospital and two neighbouring private hospitals, we attempted to identify prospectively all men and women aged 20-64 years, who were investigated by MRI because of mechanical LBP, with or without associated sciatica or neurological symptoms, during November 2003 to August 2006. These patients were informed about the study, and invited to complete a self-administered baseline questionnaire. Where a patient was investigated by MRI more than once during the study period, the invitation to participate was sent at the time of the first MRI scan. At the hospitals where the study was conducted, patients were referred for spinal MRI only by hospital specialists (mostly spinal surgeons, general orthopaedic surgeons and occasionally rheumatologists), and in comparison with LBP cases in the community, had relatively persistent symptoms with a higher prevalence of associated sciatica.

Among other things, the questionnaire addressed demographic characteristics; history of LBP and back surgery; employment history, including any change of job because of a back problem; mental health; and tendency to somatise. As part of their employment history, patients were asked whether an average working day in their current job involved any of lifting (loads > 10 kg more than ten times), working with the back bent or twisted (for longer than an hour in total), or digging/shovelling. Mental health was assessed through the relevant section of the SF-36 questionnaire [3], and was graded to three levels (good, intermediate or poor), representing approximate thirds of the distribution of scores in the study sample. Somatising tendency was assessed using questions from the Brief Symptom Inventory (BSI) [4], and was classified according to the number of symptoms from a total of five (faintness/dizziness, pain in the heart/chest, nausea/upset stomach, trouble getting breath and hot/cold spells) that had been at least moderately distressing during the past seven days. In addition, permission was sought to review the patient's MRI scan, and to send him/her a follow-up questionnaire at a later date.

MRI scans were carried out according to the protocol prescribed by the radiologist who accepted the initial referral, and were considered suitable for study if they included T1- and T2-weighted images of the lumbo-sacral spine and also axial T2-weighted images through the L3/L4, L4/L5 and L5/S1 discs. Each MRI scan was assessed by at least one of two radiologists (PM, JS) according to a standardised protocol, and classified for the presence or absence of four abnormalities - disc herniation (protrusion, extrusion or sequestration); nerve root deviation or compression; disc degeneration; and HIZ - at any of the three spinal levels from L3/L4 to L5/S1. Further details of the diagnostic criteria and the repeatability of the assessment are reported elsewhere [2].

After exclusion of patients who did not confirm LBP when questioned (4 subjects), with surgery to the back before their MRI investigation (7), and whose MRI scans could not be located (19) or were incomplete (9), 354 subjects satisfactorily completed the baseline questionnaire. Of these, 323 (91%) agreed to receive a follow-up questionnaire, and were re-contacted after an interval of approximately 22 months from the initial MRI investigation (18 months from the baseline questionnaire). Non-responders were sent a single reminder.

The follow-up questionnaire, again self-administered, asked about treatment (surgery, injections and physical therapy) since baseline, LBP and associated disability in the past four weeks, and any changes in employment since baseline because of the back problem. Disability was assessed using the Roland-Morris questionnaire [5], and LBP was classed as disabling where the score on the questionnaire exceeded 11 (this cut-point was defined before any analysis of associations with MRI findings, and was chosen to ensure adequate numbers of patients in each category). In addition, participants who were in work were asked whether during the past four weeks they had been forced to do less than their usual duties at work because of LBP.

Statistical analysis was carried out with STATA version 11 software [6]. Poisson regression was used to estimate prevalence ratios (PRs) and associated 95% confidence intervals (CIs). We first explored the relationship of patient characteristics and MRI findings to different types of treatment, and then examined risk factors for LBP and disability (including disability for work) at follow-up, adjusting for any treatment received. For this purpose, an MRI abnormality (disc herniation, nerve root deviation or compression, disc degeneration or HIZ) was classed as present if it was observed at any of the three spinal levels examined. The main aim of this analysis was to establish whether, after allowance for treatment, clinical outcomes were importantly predicted either by the number (from 0 to 4) of such abnormalities, or by the presence of any one abnormality specifically.

Ethical approval for the study was provided by the Southampton and South West Hampshire Research Ethics Committee.

## Results

Of the 323 patients who agreed to receive a follow-up questionnaire, 240 (74%) returned usable questionnaires when re-contacted. There was little difference in the response rate by sex, or according to the presence or absence of MRI abnormalities at baseline, but the response was lower at younger ages (56% in those aged less than 30 years at the time of MRI). The mean interval between MRI investigation and completion of the baseline questionnaire was 3.7 months (range 1-8 months), the mean interval from completion of the baseline questionnaire to follow-up 18.5 months (range 15-22 months), and the mean interval from MRI scan to follow-up 22.2 months (range 19-27 months). At the time when the baseline questionnaire was completed, the median duration of the current episode of LBP (i.e. the time since the patient was last free of LBP for at least one month) was 16.2 months (range 1-481 months), and 72 patients (42%) scored > 11 on the Roland-Morris questionnaire.

Table 1 summarises the demographic characteristics and MRI findings of the 240 participants, and shows also the prevalence of surgical treatment following the MRI investigation. The most common pathology on MRI was disc herniation (67% of patients), followed by HIZ (62%). Thirty-two patients (13%) had none of the four MRI abnormalities assessed, while 43 (18%) exhibited all four abnormalities. Back surgery was carried out most frequently in patients with nerve root displacement or compression (46%), and was much less common in those with none of the four MRI abnormalities (9%).

**Table 1:** Demographic characteristics of participants, MRI findings, and prevalence of surgery following MRI

			**Surgery following MRI**					
**Characteristics/abnormalities**	**No. (%) of participants**		**Before baseline questionnaire**		**After baseline questionnaire**		**Any time following MRI**	
					
			**n**	**%**	**n**	**%**	**n**	**%**
				
								
Sex								
Male	111	(46)	21	19	21	19	38	34
Female	129	(54)	24	19	20	16	36	28
								
Age at MRI (years)								
20-39	80	(33)	12	15	9	11	21	26
40-49	71	(30)	21	30	18	25	31	44
50-64	89	(37)	12	14	14	16	22	25
								
Disc degeneration								
Absent	130	(54)	26	21	19	15	39	30
Present	110	(46)	19	18	22	20	35	32
								
Disc herniation								
Absent	79	(33)	6	8	7	9	12	15
Present	161	(67)	39	25	34	21	62	39
								
Nerve root displacement/compression								
Absent	131	(55)	10	8	16	12	24	18
Present	109	(45)	35	33	25	23	50	46
								
High intensity zone								
Absent	90	(38)	20	23	15	17	30	33
Present	150	(62)	25	17	26	17	44	29
								
Number of MRI abnormalities								
0	32	(13)	1	3	2	6	3	9
1	35	(15)	5	16	4	11	7	20
2	67	(28)	15	22	14	21	26	39
3	63	(26)	13	21	9	14	20	32
4	43	(18)	11	26	12	28	18	42

Table 2 shows the prevalence of surgical and other treatment (injection or physical therapy) in the interval between the baseline and follow-up questionnaires, according to MRI findings and baseline mental health and somatising tendency. Patients who had been treated surgically between the MRI investigation and completing the baseline questionnaire were excluded from this analysis. After allowance for the number of MRI abnormalities, sex and age, back surgery tended to be more common in patients with poorer mental health at baseline (p for trend = 0.09). However, there was no corresponding trend for non-surgical treatment, and no relation to somatising tendency.

**Table 2:** Frequency of treatment during follow-up according to MRI findings and psychological characteristics at baseline

	**No surgery, injection or physical therapy**	**Surgery**			**Injection or physical therapy but not surgery**		
	**n**	**n**	**PR**^**a**^	**(95% CI)**	**n**	**PR**^**a**^	**(95% CI)**
		
							
Number of MRI abnormalities							
0	20	2	1	-	9	1	-
1	16	2	1.2	(0.2-7.8)	9	1.3	(0.6-2.9)
2	21	11	3.4	(0.8-14.3)	19	1.9	(1.0-3.6)
3	20	7	3.2	(0.7-14.3)	23	1.8	(1.0-3.3)
4	14	7	3.0	(0.7-13.0)	11	1.6	(0.8-3.2)
							
Mental Health							
Good	26	3	1	-	25	1	-
Intermediate	29	12	2.2	(0.7-7.3)	15	0.6	(0.4-1.1)
Poor	36	14	2.7	(0.8-8.9)	31	0.8	(0.5-1.3)
							
Number of distressing somatic symptoms							
0	48	17	1	-	34	1	-
1	20	6	0.8	(0.3-1.9)	17	1.1	(0.7-1.8)
≥ 2	23	6	0.8	(0.3-1.8)	20	1.2	(0.8-2.0)

^a^Prevalence ratios in comparison with those receiving none of surgery, injection or physical therapy, adjusted for the other baseline characteristics in the table, and also for sex and age (20-39, 40-49 and 50-64 years).

Analysis was restricted to the 191 patients who had not been treated surgically before completing the baseline questionnaire and who provided complete information on relevant risk factors.

At the time of follow-up, 175 patients (73%) reported LBP in the past four weeks, 89 (37%) frequent LBP (i.e. on more than 14 days in the past four weeks), and 72 (30%) disabling LBP. Table 3 shows the prevalence of these outcomes according to MRI findings, psychological risk factors at baseline, and treatment. None of the outcomes was clearly related to the number of abnormalities on the initial MRI scan. Nor was there any relation specifically to disc herniation, nerve root deviation or compression, or HIZ when analysed separately (data not shown). However, in patients with initial disc degeneration there was an increased risk of frequent (PR 1.3, 95%CI 1.0-1.9) and disabling LBP (PR 1.7, 95%CI 1.1-2.5) (Table 4). Moreover, after adjustment for treatment, disabling pain tended to be more frequent in patients who at baseline had poor mental health and a tendency to somatise (PRs for highest vs lowest categories 1.7 (95%CI 1.0-3.0) and 1.6 (95%CI 1.0-2.5) respectively when MRI findings were classified according to the number of abnormalities).

**Table 3:** Prevalence of low back pain at follow-up according to MRI findings, psychological characteristics at baseline and treatment received

	**LBP in past 4 weeks**				**LBP on > 14 days in past 4 weeks**				**Disabling LBP in past 4 weeks**^**a**^			
	**n**	**%**	**PR**^**b**^	**(95% CI)**	**n**	**%**	**PR**^**b**^	**(95% CI)**	**n**	**%**	**PR**^**b**^	**(95% CI)**
			
Number of MRI abnormalities												
0	26	81	1	-	13	41	1	-	8	25	1	-
1	22	69	0.8	(0.6-1.1)	10	31	0.6	(0.3-1.3)	9	28	0.9	(0.4-2.0)
2	45	69	0.9	(0.7-1.1)	19	30	0.7	(0.4-1.2)	17	26	0.9	(0.4-2.0)
3	44	72	0.9	(0.7-1.1)	25	43	0.9	(0.6-1.6)	16	26	0.9	(0.4-1.9)
4	32	74	1.0	(0.8-1.2)	18	43	1.0	(0.6-1.8)	20	47	1.8	(0.9-3.6)
												
Mental Health												
Good	48	68	1	-	21	30	1	-	14	20	1	-
Intermediate	55	75	1.2	(0.9-1.4)	27	39	1.4	(0.9-2.2)	23	32	1.6	(0.9-2.9)
Poor	66	74	1.1	(0.9-1.3)	37	42	1.3	(0.8-2.1)	33	37	1.7	(0.9-3.0)
												
Number of distressing somatic symptoms												
0	88	68	1	-	41	33	1	-	31	24	1	-
1	36	72	1.1	(0.9-1.3)	17	36	1.0	(0.6-1.6)	17	34	1.4	(0.8-2.3)
≥2	45	83	1.2	(1.0-1.5)	27	50	1.4	(0.9-2.1)	22	41	1.6	(1.0-2.5)
												
Treatment												
None^c^	77	69	1	-	35	32	1	-	32	29	1	-
Surgery^d^	23	56	0.8	(0.6-1.1)	12	30	0.9	(0.5-1.6)	10	24	0.7	(0.4-1.4)
Injections or physical therapy but not surgery^e^	69	85	1.2	(1.1-1.4)	38	49	1.6	(1.1-2.3)	28	35	1.3	(0.8-1.9)

^a^Roland Morris score > 11

^b^Prevalence ratios adjusted for the other risk factors in the table, and also for sex and age (20-39, 40-49 and 50-64 years). For each regression model, analysis was restricted to patients with complete information on all relevant variables (233 for LBP in past 4 weeks, 227 for LBP on > 14 days in past 4 weeks, and 233 for disabling LBP in past 4 weeks).

^c^No back surgery after MRI investigation and no injections or physical therapy after baseline questionnaire

^d^Back surgery after MRI investigation

^e^Injections or physical therapy after baseline questionnaire, but no back surgery after MRI investigation

**Table 4:** Prevalence of low back pain at follow-up according to presence of disc degeneration, psychological characteristics at baseline and treatment received.

	**LBP in past 4 weeks**				**LBP on > 14 days in past 4 weeks**				**Disabling LBP in past 4 weeks**^**a**^			
	**n**	**%**	**PR**^**b**^	**(95% CI)**	**n**	**%**	**PR**^**b**^	**(95% CI)**	**n**	**%**	**PR**^**b**^	**(95% CI)**
			
												
Disc degeneration												
Absent	86	70	1	-	39	32	1	-	28	23	1	-
Present	83	75	1.1	(1.0-1.3)	46	43	1.3	(1.0-1.9)	42	38	1.7	(1.1-2.5)
												
Mental Health												
Good	48	68	1	-	21	30	1	-	14	20	1	-
Intermediate	55	75	1.1	(0.9-1.4)	27	39	1.3	(0.8-2.1)	23	32	1.6	(0.9-2.8)
Poor	66	74	1.1	(0.9-1.3)	37	42	1.2	(0.8-2.0)	33	37	1.7	(1.0-3.0)
												
Number of distressing somatic symptoms												
0	88	68	1	-	41	33	1	-	31	24	1	-
1	36	72	1.1	(0.9-1.3)	17	36	1.1	(0.7-1.7)	17	34	1.3	(0.8-2.2)
≥2	45	83	1.2	(1.0-1.5)	27	50	1.5	(1.0-2.2)	22	41	1.6	(1.0-2.5)
												
Treatment												
None^c^	77	69	1	-	35	32	1	-	32	29	1	-
Surgery^d^	23	56	0.8	(0.6-1.1)	12	30	0.9	(0.5-1.5)	10	24	0.7	(0.4-1.4)
Injections or physical therapy but not surgery^e^	69	85	1.2	(1.0-1.4)	38	49	1.5	(1.1-2.2)	28	35	1.2	(0.8-1.8)

^a^Roland Morris score > 11

^b^Prevalence ratios adjusted for the other risk factors in the table, and also for sex and age (20-39, 40-49 and 50-64 years). For each regression model, analysis was restricted to patients with complete information on all relevant variables (233 for LBP in past 4 weeks, 227 for LBP on > 14 days in past 4 weeks, and 233 for disabling LBP in past 4 weeks).

^c^No back surgery after MRI investigation and no injections or physical therapy after baseline questionnaire

^d^Back surgery after MRI investigation

^e^Injections or physical therapy after baseline questionnaire, but no back surgery after MRI investigation

Among 170 patients who held a job at the time of the baseline questionnaire, 20 (12%) subsequently left that job because of their back problem, and of the 128 who were still in the same job at the time of follow-up, 30 (23%) had been unable to carry out their normal duties at work in the four weeks before answering the follow-up questionnaire. These adverse employment outcomes were more frequent in patients treated surgically or by injection or physical therapy (Table 5). After adjustment for treatment, they were also more common in patients whose work at baseline entailed lifting, bending, twisting, digging or shovelling (PR 1.8, 95%CI 1.1-3.0). However, there was no clear relation to number of MRI abnormalities, or to baseline mental health or somatising tendency. Nor were they associated with any specific MRI abnormality (Tables 6, 7, 8 and 9).

**Table 5:** Employment outcomes at follow-up according to MRI findings, psychological characteristics and occupational activities at baseline, and treatment received.

	**Job left or work restricted because of LBP**^**a**^			
	**n**	**%**	**PR**^**b**^	**(95% CI)**
	
				
Number of MRI abnormalities				
0	8	32	1	-
1	7	33	0.9	(0.4-2.1)
2	11	26	0.6	(0.3-1.3)
3	11	24	0.6	(0.3-1.2)
4	10	34	0.9	(0.4-1.8)
				
Mental health				
Good	14	23	1	-
Intermediate	16	33	1.3	(0.7-2.4)
Poor	17	32	1.2	(0.6-2.4)
				
Number of distressing somatic symptoms				
0	24	24	1	-
1	9	26	0.9	(0.5-1.6)
≥ 2	14	45	1.4	(0.8-2.5)
				
Job at baseline entailed lifting, bending, twisting, digging or shovelling^c^				
No	26	22	1	-
Yes	21	46	1.8	(1.1-3.0)
				
Treatment				
None^d^	15	19	1	-
Surgery^e^	13	45	2.3	(1.2-4.5)
Injection or physical therapy but not surgery^f^	19	35	1.9	(1.1-3.4)

^a^Job left because of back problem in interval between baseline and follow-up questionnaires, or still in same job at time of answering the follow-up questionnaire but with restriction of normal work activities in the past four weeks. Analysis was restricted to the 164 patients who held a job when they completed the baseline questionnaire and who provided complete information on relevant risk factors.

^b^Prevalence ratios adjusted for the other risk factors in the table and also for sex and age (20-39, 40-49 and 50-64 years)

^c^An average working day entailed lifting loads > 10 kg more than ten times, working with the back bent or twisted for longer than an hour, or digging/shovelling

^d^No back surgery after MRI investigation and no injections or physical therapy after baseline questionnaire

^e^Back surgery after MRI investigation

^f^Injections or physical therapy after baseline questionnaire, but no back surgery after MRI investigation

**Table 6:** Employment outcomes at follow-up according to presence of disc degeneration on MRI, psychological characteristics and occupational activities at baseline, and treatment received

	**Job left or work restricted because of LBP**^**a**^			
	**n**	**%**	**PR**^**b**^	**(95% CI)**
	
				
Disc degeneration				
Absent	23	26	1	-
Present	24	31	1.2	(0.8-2.0)
				
Mental health				
Good	14	23	1	-
Intermediate	16	33	1.2	(0.7-2.2)
Poor	17	32	1.2	(0.6-2.3)
				
Number of distressing somatic symptoms				
0	24	24	1	-
1	9	26	0.9	(0.5-1.7)
≥ 2	14	45	1.4	(0.8-2.7)
				
Job at baseline entailed lifting, bending, twisting, digging or shovelling^c^				
No	26	22	1	-
Yes	21	46	1.9	(1.1-3.0)
				
Treatment				
None^d^	15	19	1	-
Surgery^e^	13	45	2.2	(1.2-4.0)
Injection or physical therapy but not surgery^f^	19	35	1.8	(1.0-3.1)

^a^Job left because of back problem in interval between baseline and follow-up questionnaires, or still in same job at time of answering the follow-up questionnaire but with restriction of normal work activities in the past four weeks. Analysis was restricted to the 164 patients who held a job when they completed the baseline questionnaire and who provided complete information on relevant risk factors.

^b^Prevalence ratios adjusted for the other risk factors in the table and also for sex and age (20-39, 40-49 and 50-64 years)

^c^An average working day entailed lifting loads > 10 kg more than ten times, working with the back bent or twisted for longer than an hour, or digging/shovelling

^d^No back surgery after MRI investigation and no injections or physical therapy after baseline questionnaire

^e^Back surgery after MRI investigation

^f^Injections or physical therapy after baseline questionnaire, but no back surgery after MRI investigation

**Table 7:** Employment outcomes at follow-up according to presence of disc herniation on MRI, psychological characteristics and occupational activities at baseline, and treatment received

	**Job left or work restricted because of LBP**^**a**^			
	**n**	**%**	**PR**^**b**^	**(95% CI)**
	
				
Disc herniation				
Absent	17	31	1	-
Present	30	28	0.7	(0.4-1.2)
				
Mental health				
Good	14	23	1	-
Intermediate	16	33	1.3	(0.7-2.3)
Poor	17	32	1.3	(0.7-2.4)
				
Number of distressing somatic symptoms				
0	24	24	1	-
1	9	26	0.9	(0.5-1.6)
≥ 2	14	45	1.4	(0.7-2.6)
				
Job at baseline entailed lifting, bending, twisting, digging or shovelling^c^				
No	26	22	1	-
Yes	21	46	1.8	(1.1-3.0)
				
Treatment				
None^d^	15	19	1	-
Surgery^e^	13	45	2.4	(1.3-4.7)
Injection or physical therapy but not surgery^f^	19	35	2.0	(1.1-3.5)

^a^Job left because of back problem in interval between baseline and follow-up questionnaires, or still in same job at time of answering the follow-up questionnaire but with restriction of normal work activities in the past four weeks. Analysis was restricted to the 164 patients who held a job when they completed the baseline questionnaire and who provided complete information on relevant risk factors.

^b^Prevalence ratios adjusted for the other risk factors in the table and also for sex and age (20-39, 40-49 and 50-64 years)

^c^An average working day entailed lifting loads > 10 kg more than ten times, working with the back bent or twisted for longer than an hour, or digging/shovelling

^d^No back surgery after MRI investigation and no injections or physical therapy after baseline questionnaire

^e^Back surgery after MRI investigation

^f^Injections or physical therapy after baseline questionnaire, but no back surgery after MRI investigation

**Table 8:** Employment outcomes at follow-up according to presence of nerve root displacement/compression on MRI, psychological characteristics and occupational activities at baseline, and treatment received

	**Job left or work restricted because of LBP**^**a**^			
	**n**	**%**	**PR**^**b**^	**(95% CI)**
	
				
Nerve root displacement/compression				
Absent	26	30	1	-
Present	21	28	0.9	(0.6-1.5)
				
Mental health				
Good	14	23	1	-
Intermediate	16	33	1.2	(0.7-2.2)
Poor	17	32	1.2	(0.6-2.3)
				
Number of distressing somatic symptoms				
0	24	24	1	-
1	9	26	0.9	(0.5-1.7)
≥ 2	14	45	1.4	(0.8-2.6)
				
Job at baseline entailed lifting, bending, twisting, digging or shovelling^c^				
No	26	22	1	-
Yes	21	46	1.8	(1.1-2.9)
				
Treatment				
None^d^	15	19	1	-
Surgery^e^	13	45	2.2	(1.2-4.3)
Injection or physical therapy but not surgery^f^	19	35	1.8	(1.1-3.2)

^a^Job left because of back problem in interval between baseline and follow-up questionnaires, or still in same job at time of answering the follow-up questionnaire but with restriction of normal work activities in the past four weeks. Analysis was restricted to the 164 patients who held a job when they completed the baseline questionnaire and who provided complete information on relevant risk factors.

^b^Prevalence ratios adjusted for the other risk factors in the table and also for sex and age (20-39, 40-49 and 50-64 years)

^c^An average working day entailed lifting loads > 10 kg more than ten times, working with the back bent or twisted for longer than an hour, or digging/shovelling

^d^No back surgery after MRI investigation and no injections or physical therapy after baseline questionnaire

^e^Back surgery after MRI investigation

^f^Injections or physical therapy after baseline questionnaire, but no back surgery after MRI investigation

**Table 9:** Employment outcomes at follow-up according to presence of HIZ on MRI, psychological characteristics and occupational activities at baseline, and treatment received

	**Job left or work restricted because of LBP**^**a**^			
	**n**	**%**	**PR**^**b**^	**(95% CI)**
	
				
HIZ				
Absent	20	31	1	-
Present	27	27	0.8	(0.5-1.3)
				
Mental health				
Good	14	23	1	-
Intermediate	16	33	1.2	(0.7-2.2)
Poor	17	32	1.2	(0.6-2.4)
				
Number of distressing somatic symptoms				
0	24	24	1	-
1	9	26	0.9	(0.5-1.7)
≥ 2	14	45	1.4	(0.8-2.6)
				
Job at baseline entailed lifting, bending, twisting, digging or shovelling^c^				
No	26	22	1	-
Yes	21	46	1.8	(1.1-2.9)
				
Treatment				
None^d^	15	19	1	-
Surgery^e^	13	45	2.2	(1.2-4.2)
Injection or physical therapy but not surgery^f^	19	35	1.9	(1.1-3.3)

^a^Job left because of back problem in interval between baseline and follow-up questionnaires, or still in same job at time of answering the follow-up questionnaire but with restriction of normal work activities in the past four weeks. Analysis was restricted to the 164 patients who held a job when they completed the baseline questionnaire and who provided complete information on relevant risk factors.

^b^Prevalence ratios adjusted for the other risk factors in the table and also for sex and age (20-39, 40-49 and 50-64 years)

^c^An average working day entailed lifting loads > 10 kg more than ten times, working with the back bent or twisted for longer than an hour, or digging/shovelling

^d^No back surgery after MRI investigation and no injections or physical therapy after baseline questionnaire

^e^Back surgery after MRI investigation

^f^Injections or physical therapy after baseline questionnaire, but no back surgery after MRI investigation

## Discussion

Among the patients whom we studied, surgical treatment was more common in those with multiple MRI abnormalities, and especially with nerve root displacement or compression. Moreover, disc degeneration on MRI was associated with a higher prevalence of frequent and disabling LBP at follow-up some 22 months later. In general, however, pathology on MRI showed little relationship to later symptoms or disability. Disabling LBP was more strongly predicted by poor mental health and tendency to somatise, while adverse employment outcomes depended more on the physical demands of the patient's job.

Of necessity, our study was limited to patients who had been referred to participating radiology departments for investigation of their back symptoms. As such, they will not have been representative of all LBP patients in the severity and persistence of their symptoms or their clinical outcomes. However, it seems unlikely that the MRI abnormalities investigated would importantly predict clinical outcomes in the generality of people with LBP when they failed to do so in the sample of patients whom we studied.

For practical reasons, participants could only be contacted after their initial MRI investigation, and by the time they completed the baseline questionnaire some had already undergone surgery. In addition, some are likely already to have received other forms of treatment during their LBP episode, either before or after the MRI scan. As a consequence, it is not possible to exclude an influence of earlier treatment on mental health and somatising tendency as assessed at baseline. Nevertheless, controlling for treatment as we did, enabled more meaningful assessment of associations with these prognostic variables.

We restricted our analyses to outcomes that post-dated the risk factors under consideration. In some cases, therefore, the data presented do not fully convey the impact of LBP on participating patients. For example, in addition to the 20 patients who left the job they held at baseline because of their back problem, nine had given up a job between the MRI scan and completing the baseline questionnaire, and others may have changed employment even earlier in the course of the illness.

Another limitation was our reliance on patient recall for ascertainment of most of the variables analysed. Thus, among the 45 subjects who in the baseline questionnaire reported surgical treatment following their MRI scan, 12 indicated at follow-up that they had been treated surgically in the interval since the baseline questionnaire (Table 1). While some of these patients may have undergone more than one operation, it is also possible that both reports referred to the same operation, but that its exact timing in relation to the baseline questionnaire was not remembered accurately at follow-up. However, for most of the variables studied, assessment depended only on recent recall, and therefore should have been more reliable.

A further potential source of error was inaccurate classification of MRI abnormalities. As described elsewhere, care was taken to standardise the reading of MRI scans, and repeatability within and between the two observers was generally good [2]. Moreover, the assessments were carried out without knowledge of patients' clinical histories or of the information that they had provided in the baseline questionnaire. It is, of course, possible that patients had spinal abnormalities other than those assessed (e.g. facet joint arthritis), or at other spinal levels. However, the abnormalities investigated were those which have been related most consistently to LBP [1], and the spinal levels studied were those at which causes for LBP are most frequently found.

That surgical treatment was more common in patients with spinal pathology on MRI is unsurprising. More remarkable is that surgery was performed in three (9%) of the patients who had none of the MRI abnormalities that we assessed. This may have been because they had other forms of pathology (including lesions at levels other than the three that we examined), or because the MRI scan was interpreted differently by clinicians who were caring for the patient. In addition, there was some indication that surgery was performed more frequently in patients with poor mental health. This may reflect a greater desire of such patients to undergo surgery, or greater willingness of surgeons to operate on patients who appear more distressed.

None of the four MRI abnormalities that we assessed carried a higher overall risk of LBP at follow-up, although frequent and disabling pain was more common in patients with initial evidence of disc degeneration. Other studies looking at the prognosis of LBP in relation to spinal pathology have produced inconsistent results. Among a group of patients treated by spinal fusion for chronic LBP, low disc height was associated with functional improvement after two years [7]. On the other hand, in follow-up of patients treated by epidural steroid injections for lumbosacral disc prolapse, poor disc hydration was not significantly related to subsequent levels of pain and satisfaction, but outcomes were worse in those with higher grades of nerve root compression [8]. In a French investigation, patients with new sciatica or femoral neuralgia that was managed conservatively tended to improve more if nerve root compression was observed on baseline computerised tomography, but outcome was unrelated to features of disc herniation [9]. However, a study of "active conservative" management for sciatica found higher rates of recovery in patients with disc extrusions or broad-based protrusions, but no clear difference in recovery according to whether there was root compromise [10]. Against this, among patients with acute radicular pain and lumbar disc prolapse or protrusion, persistence of pain was predicted by the degree of disc displacement [11], and in follow-up of a cohort of patients with sciatica who were managed without surgery, outcomes were better when the ratio of disc hernia area to remaining canal area was small [12]. Thus, there was no prior expectation of continuing frequent and disabling LBP in patients with disc degeneration as opposed to other spinal pathology, and the observed relationship may have occurred simply by chance.

Disabling LBP at follow-up was also predicted by poor mental health and tendency to somatise at baseline. Most other studies have also found that poor mental health and/or somatising tendency were associated with worse outcomes in patients with LBP [11,13-20], and while the finding has not been universal [7], a systematic review concluded that both depressed mood and somatisation are implicated in the chronicity of LBP [21].

Adverse employment outcomes were associated with treatment by surgery, injection or physical therapy. We cannot be sure that job loss during follow-up always post-dated these treatments. However, patients with LBP of sufficient severity to warrant more active treatment might be expected to have a worse prognosis. Nor is it surprising that patients with physically demanding work are more likely to have difficulty in carrying out their normal duties and in retaining their jobs, although the high frequency of such limitations that we observed among those with physically demanding jobs (46%) is notable. At the same time, we found no indication that MRI abnormalities importantly predicted employment outcomes. The relationship of MRI findings to subsequent employment has been little studied previously, but in a cohort study of people with asymptomatic disc herniation, physical job characteristics and psychological aspects of work were stronger predictors of future need for medical consultation and resultant work incapacity than MRI-identified disc abnormalities [22].

## Conclusions

In epidemiological research, LBP is often treated as a single diagnostic entity, but in theory there might be advantages in distinguishing cases that are associated with specific pathology in the spine. This would apply if the sub-categories of LBP so defined differed importantly in their associations with risk factors or in their prognosis. In a previously reported analysis, we found no evidence that the presence of MRI abnormalities distinguished a subset of LBP with different risk factors [2]. The data presented here suggest that MRI abnormalities also have little prognostic value for symptoms and disability, although they may determine the way in which a patient is treated. We thus find no support for refining case definition of LBP in epidemiological research according to the presence or absence of such MRI abnormalities.

## Competing interests

The authors declare that they have no competing interests.

## Authors' contributions

PM and JS reviewed and classified the MRI scans. ECH managed all other aspects of the data collection. MK carried out the statistical analysis. MS oversaw the MRI classification. KTP and DC conceived and designed the study. DC wrote the first draft of the report. All of the authors reviewed and commented on the report, and approved the final version.

## Pre-publication history

The pre-publication history for this paper can be accessed here:

http://www.biomedcentral.com/1471-2474/12/234/prepub
